# Reversing regulatory safeguards: Targeting the ATR pathway to overcome PARP inhibitor resistance

**DOI:** 10.1016/j.omton.2025.200934

**Published:** 2025-01-14

**Authors:** Xixi Lin, Ye Qiu, Aashish Soni, Martin Stuschke, George Iliakis

**Affiliations:** 1 Department of Radiation Therapy, Division of Experimental Radiation Biology, University Hospital Essen, University of Duisburg-Essen, 45147 Essen, Germany; 2 Institute of Medical Radiation Biology, University Hospital Essen, University of Duisburg-Essen, 45147 Essen, Germany; 3 German Cancer Consortium (DKTK), Partner Site University Hospital Essen, German Cancer Research Center (DKFZ), 45147 Essen, Germany

**Keywords:** MT: Regular Issue, PARP inhibitors, ATR inhibitors, homologous recombination, chemotherapy, cancer, DNA double-stranded breaks, replication fork, cell-cycle checkpoint

## Abstract

The development of poly (ADP-ribose) polymerase inhibitors (PARPis) is widely considered a therapeutic milestone in the management of BRCA1/2-deficient malignancies. Since a growing number of cancer treatment guidelines include PARPis, the inevitably emerging PARPi resistance becomes a serious limitation that must be addressed. Targeting the DNA damage response signaling kinase, ATR (ataxia telangiectasia and rad3-related serine/threonine kinase), activated in response to PARPi-induced replication stress, represents a promising approach in fighting PARPi-resistant cancers. The success of this combination therapy in preclinical models has inspired efforts to translate its potential through extensive clinical research and clinical trials. However, the available clinical evidence suggests that PARPi/ATRi combinations have yet to reach their anticipated therapeutic potential. In this review, we summarize work elucidating mechanisms underpinning the effectiveness of ATRi in fighting PARPi resistance and review translational studies reporting efficacy in different types of cancer. Finally, we discuss potential biomarkers of patient selection for customized combinations of PARPi/ATRi treatments.

## Graphical abstract

**Figure:**
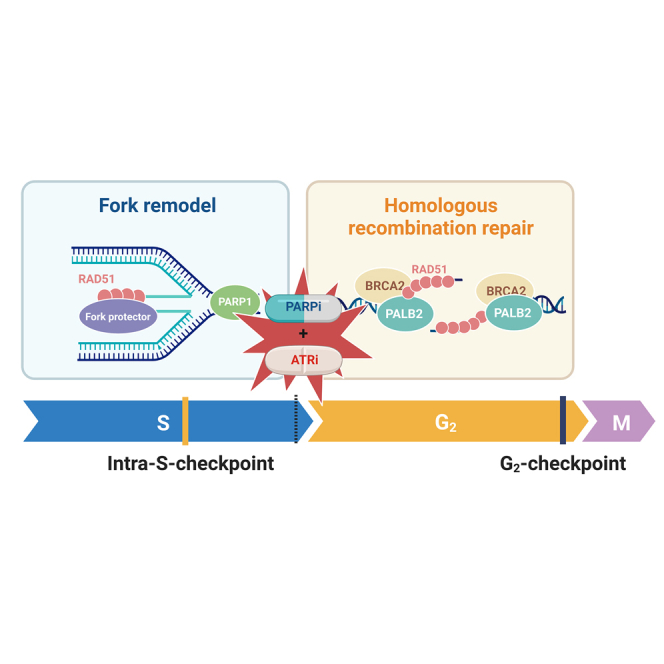

## Teaser

Iliakis and colleagues review the potential of ATR inhibitors as a strategy to overcome PARPi resistance. The article highlights preclinical and clinical observations supporting this strategy and discusses biomarkers for optimizing PARPi/ATRi-combination therapies.

## Introduction

Over the past decade, the landscape of targeted cancer therapies has been enriched by the development and regulatory approval of poly (ADP-ribose) polymerase inhibitors (PARPis), and several PARPis have been integrated into therapeutic regimens for a wide spectrum of malignancies.^1^^,^^2^^,^^3^^,^^4^^,^^5^ Functionally, contemporary PARPis are designed to mimic nicotinamide adenine dinucleotide, thereby inhibiting the enzymatic activity of PARP1. This inhibition is pivotal, as PARP1 plays a role in the repair of single-stranded DNA (ssDNA) breaks (SSBs) and helps them to maintain genomic stability and cell viability.^1^ Mechanistically, PARPis trap PARP1 proteins recruited to sites of SSBs, forming non-covalent PARP1-DNA adducts that impede replication fork progression.^6^^,^^7^^,^^8^ Alternatively, replisomes within forks collide with the transcription-replication conflicts resulting from the inhibition of PARP1 enzymatic activity by PARPi.^9^ Both scenarios lead to the formation of DNA double-stranded breaks (DSBs) that rely on homologous recombination (HR) for accurate repair.^10^^,^^11^ Indeed, BRCA mutation is the HR-deficient (HRD) genetic background where the efficacy of PARPis was originally documented.^10^^,^^12^ This form of “synthetic lethality” provides a proof of concept for the development of clinical approaches, ushering oncology into the era of individualized, mechanism-based cancer therapy. Olaparib paved the way for the clinical application of PARPi and was the first compound to receive US Food and Drug Administration approval in 2014 as a maintenance treatment for advanced ovarian cancer (OC) with germline breast cancer gene 1/2 (BRCA1/2) mutations in patients with three or more unsuccessful lines of chemotherapy. Subsequently, other PARPis, such as rucaparib, niraparib, and talazoparib, received regulatory approval for various indications in ovarian, breast, prostate, and pancreatic cancer.^1^^,^^2^

While PARPis have significantly shifted treatment paradigms in tumors with HRD, the emergence of resistance remains a significant hurdle. This resistance often involves complex biological adaptations, including the restoration of replication fork stability and HR, which counteract the therapeutic effects of PARPis.^13^ Such developments necessitate research to understand and overcome resistance mechanisms to enhance the curability of PARPi-based therapies. PARP1 is implicated in fork protection and cooperates with BRCA2 to protect forks from meiotic recombination 11 (MRE11)-dependent degradation.^14^ PARP1 also plays a crucial role in ligating Okazaki fragments within replication forks, thereby promoting fork stability.^15^ Consequently, tumors subjected to PARPi often exhibit signs of slowing or stalling of replicons to generate replication stress.^16^^,^^17^

The manifestation of replication stress triggers a response primarily governed by the ataxia telangiectasia and rad3-related serine/threonine kinase (ATR), a central checkpoint protein kinase. Activation of ATR inhibits cell-cycle progression to prevent mitotic catastrophe and locally stabilizes replication forks,^18^^,^^19^ thereby compromising PARPi efficacy. Furthermore, ATR is implicated in HR processes by coordinating the activities of HR factors such as BRCA1, partner and localizer of BRCA2 (PALB2), and RAD51 paralogs in the post-resection stage.^20^^,^^21^^,^^22^ The inhibition of ATR has been shown to induce an HRD status, underscoring its potential as a therapeutic target in cancer treatments.^23^^,^^24^ These biological processes are intricately interconnected within a complex, kinase-driven signaling network called the DNA damage response (DDR). Insights into the molecular dynamics of the DDR offer a theoretical framework for targeting ATR as a strategy to counteract PARPi resistance. This review outlines our current understanding of the molecular mechanisms contributing to PARPi resistance and explores the potential benefits of combining PARPi with ATR inhibitors (ATRis). We specifically focus on the integration of these strategies into the DDR framework, highlighting approaches that are currently tested for efficacy in clinical trials.

### Roles of ATR in PARPi resistance

#### DNA replication fork protection

Restoration of stalled-fork stabilization is recognized as a major contributor to PARPi resistance that operates independently of HR restoration. In standard cancer therapy protocols, DNA-damaging agents, such as cisplatin, in combination with PARPis induce DNA replication stress. This stress manifests as a slowing, stalling, or collapse of the replication fork during DNA synthesis.^25^^,^^26^ When damage occurs on the leading strand template, the resulting fork stalling leads to a decoupling from DNA synthesis of helicase-mediated DNA unwinding. This decoupling produces extended regions of ssDNA, which are vulnerable to nucleolytic degradation and can ultimately cause genetic rearrangements.^14^^,^^25^^,^^27^ To maintain the stability of DNA replication in the face of such events, cells utilize several DNA damage tolerance (DDT) mechanisms. One mechanism of DDT is to bypass the lesion, either using translesion synthesis or repriming of DNA synthesis after the lesion.^28^^,^^29^ Another mechanism is fork remodeling, which generates a stable fork structure to allow engagement of the DNA repair machinery that removes the lesion and prevents DSB formation.^30^ DNA replication fork dynamics are now central in research designed to unravel the mechanisms of PARPi resistance.

ssDNA regions generated during replication stress caused by PARPi are covered by replication protein A (RPA) that in turn recruits ATR through its DNA interacting protein subunit ATRIP. Many ATR functions are affected by its downstream effector checkpoint kinase 1 (CHK1), including signaling and recovery of compromised DNA replication.^19^

**Figure 1:**
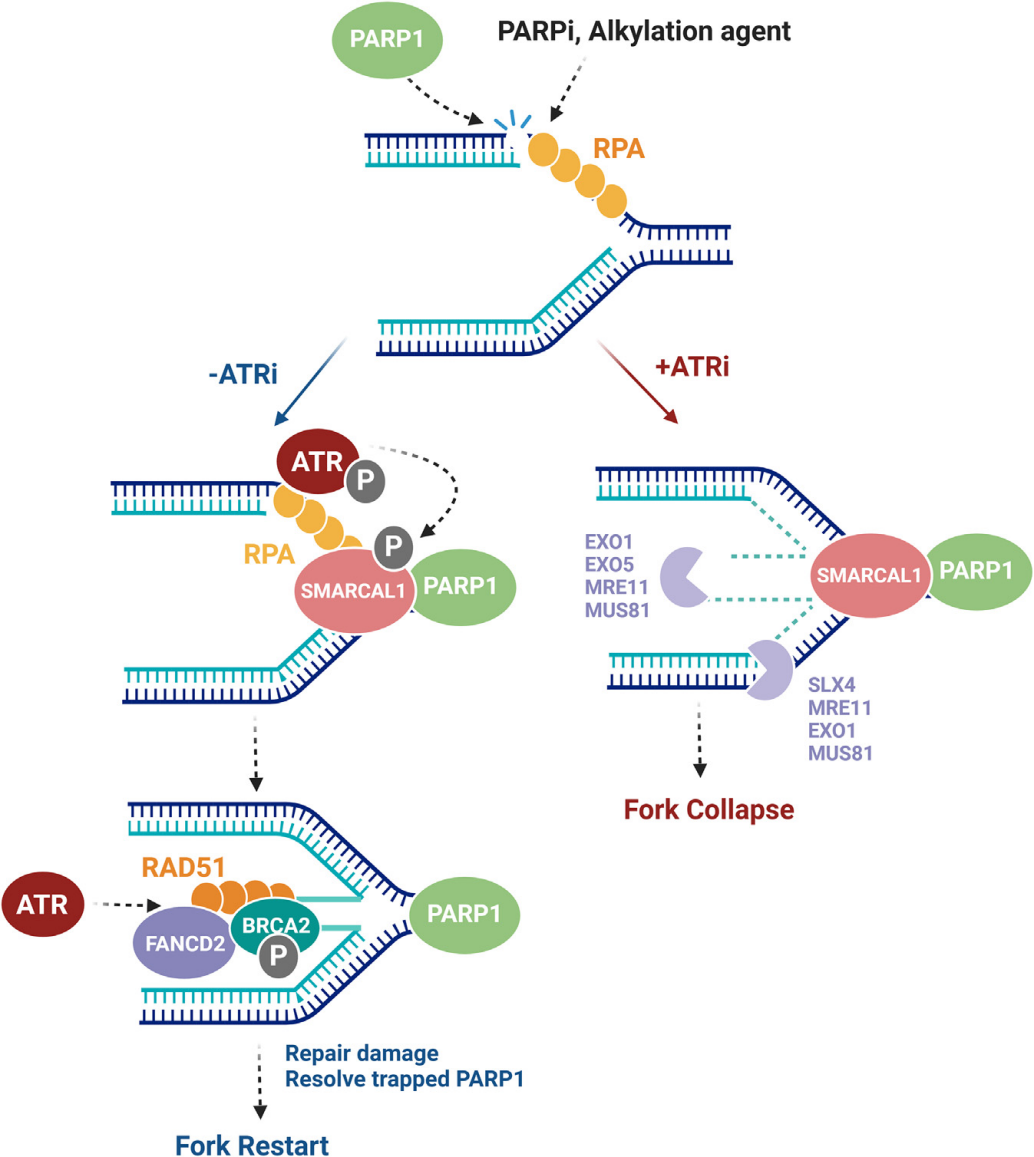
ATR protects replication fork upon PARPi treatment The treatment with PARPi in combination with alkylating agents triggers replication stress, as the replication fork stalls at the site of the PARP-trapping lesion and undergoes uncoupling from helicase-induced unwinding. RPA promptly binds to the resulting, exposed ssDNAs, recruiting fork remodeler SMARCAL1 and activating ATR. SMARCAL1 facilitates fork reversal via ATR phosphorylation. Subsequently, BRCA2 relocates to the reversed fork and promotes the formation of RAD51 nucleofilaments. Fork protectors such as FANCD2 are recruited to the reversed fork protecting against nucleolytic degradation. ATR supports activities involved in this process, which facilitates DNA repair and fork restart. When ATR is inhibited, the fork structure, including nascent and template strands, becomes susceptible to degradation by various nucleases (e.g., EXO1, EXO5, MRE11, MUS81, SLX4), inevitably leading to fork collapse. (The figure was created in BioRender).

ATR protects DNA replication forks by different mechanisms. One key mechanism of DNA replication fork protection is fork remodeling. When replication forks encounter DNA lesions or obstacles on the template strand (e.g., trapped PARP1), they typically undergo stabilization through the reversal and annealing of the two nascent strands, forming four-way DNA junctions, referred to as reversed forks or “chicken feet.”^30^ While fork reversal is beneficial for stabilizing and facilitating fork recovery under mild replication stress, the formation of the forks also increases the probability of excessive nuclease cleavage that increases the load of DSBs, particularly under conditions of high replication stress or in absence of BRCA1/2.^25^^,^^31^ In this context, ATR was shown to phosphorylate the chromatin remodeler SMARCAL1, which interacts with RPA and catalyzes fork reversal, thereby suppressing DSB formation by SLX4-dependent nucleases.^32^^,^^33^ After fork reversal, BRCA2 is recruited to stalled DNA replication forks to promote the formation of RAD51 nucleoprotein filaments.^34^ These nucleofilaments serve as a platform for the recruitment of other fork protectors such as Fanconi anemia complementation group (FANC) B and FANCD2 that suppress fork degradation.^35^ During this process, ATR regulates the phosphorylation of BRCA2 through large tumor suppressor kinase 1-cyclin-dependent kinase 2 (CDK2) interactions and supports RAD51 nucleofilament formation.^36^ Moreover, ATR promotes FANCD2 loading by phosphorylating mini-chromosome maintenance complex component 2–7 helicases^37^ (Figure 1).

Another mechanism by which ATR protects stressed DNA replication forks is to prevent excessive cleavage by various nucleases. For example, ATR phosphorylates exonuclease 1 (EXO1), promoting its ubiquitylation-dependent degradation, thus minimizing its activity on stalled replication forks.^38^^,^^39^ ATR also acts on EXO5, a distinct exonuclease analogous to DNA2, forming an EXO5-DNA structure that directs fork restart in coordination with Bloom syndrome protein (BLM) helicase.^40^ Additionally, when stalled replication forks collide with DNA transcription, ATR signaling is activated by R-loops and the MUS81 nuclease, which suppresses in turn excessive MUS81 cleavage and enables replication fork recovery^41^ (Figure 1). Significantly, greater tumor cell killing was observed in patient-derived organoids exhibiting unstable DNA replication fork phenotypes upon treatment with ATRi.^42^

A recent study validated the protective roles of ATR in DNA replication using different DNA labeling assays. It revealed that ATR suppresses fork uncoupling and MRE11/EXO1-mediated degradation of both nascent and template DNA strands. Additionally, ATR inhibition can overcome restored ssDNA gap protection at ongoing DNA replication forks in BRCA1-deficient, PARPi-resistant cells.^26^ In summary, ATR emerges as a master regulator of the DNA replication stress response, safeguarding DNA replication fork progression during PARP inhibition and enforcing cell tolerance to PARPi treatment.

#### Restoration of HR repair and DNA end resection

While DNA replication fork protection stands out as an emerging PARPi resistance mechanism, the restoration of HR has long been recognized as a pivotal resistance mechanism. HR restoration in a BRCA-deficient genetic background includes reversion mutations that restore BRCA function and epigenetic upregulation of BRCA1/2 that increases the ability of cells to carry out HR. BRCA-independent pathways of HR restoration include the loss of negative regulators of HR (e.g., 53BP1, or the shieldin complex), and reversion mutations in HR genes, such as RAD51 paralogs or PALB2. Detailed descriptions of the above PARPi resistance mechanisms have been extensively reviewed (for example, in Dias et al.^13^). The present review focuses therefore on the role of ATR in PARPi-treated cells with functional HR.

ATR is an established regulator of HR, a DSB repair pathway operating during S/G_2_ phases of the cell cycle and relying on the homologous template present in the sister chromatid. HR is initiated by DNA end resection, generating 3′-ssDNA overhangs, subsequently coated by RPA. ATR is activated on this structure through ATRIP and DNA topoisomerase 2-binding protein 1 (TOPBP1),^43^^,^^44^ leading to the phosphorylation of H2AX and the recruitment of BRCA1. This sets the stage for the recruitment of the PALB2-BRCA2 complex to facilitate RAD51-ssDNA filament formation and thus homology searching and strand invasion.^11^ During these processes, ATR phosphorylates PALB2 at the S59 site, enhancing its localization to DNA lesions via interactions with BRCA1.^45^ ATR also directly increases the phosphorylation of RPA32, BRCA1, and RAD51 paralogs.^20^^,^^21^^,^^22^

**Figure 2:**
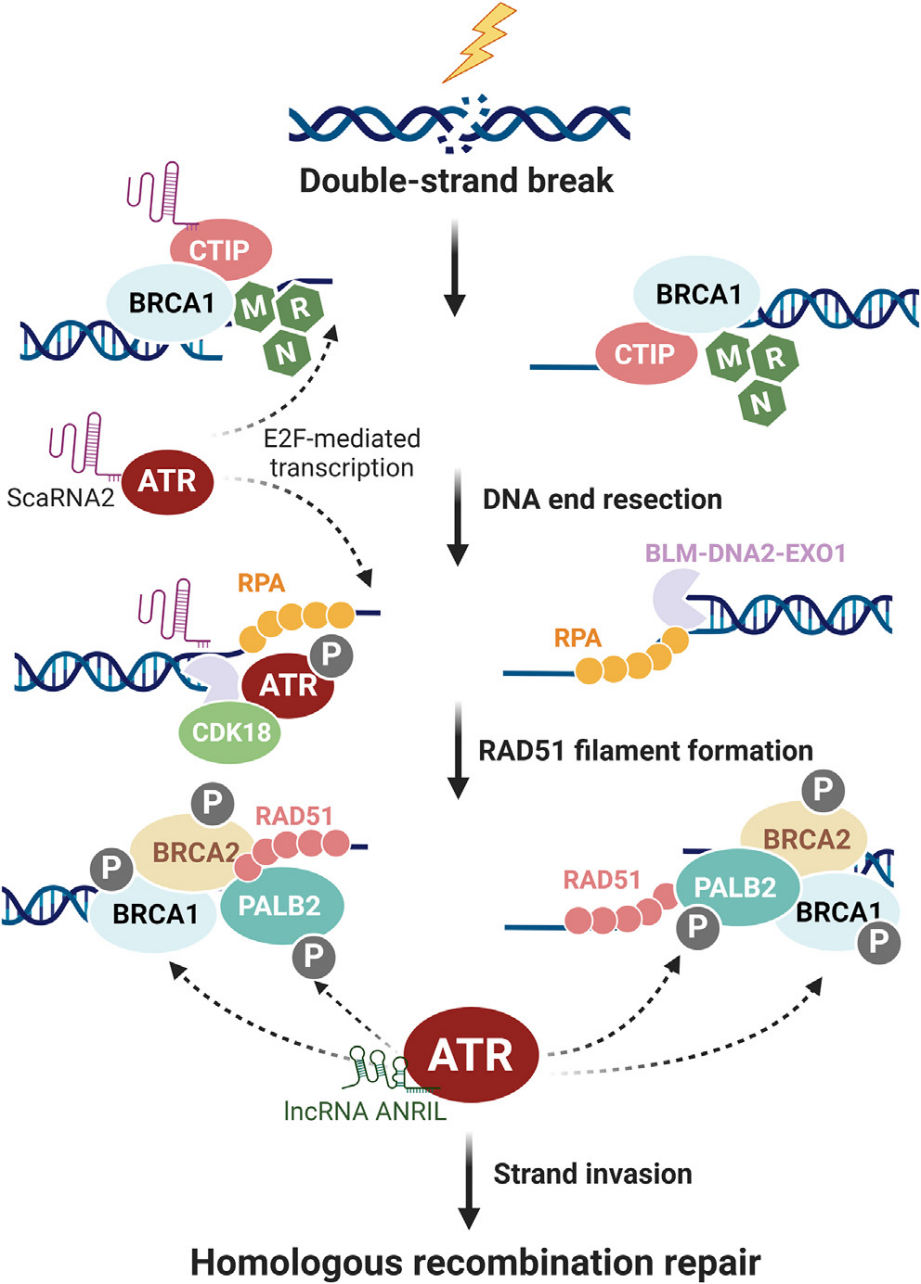
ATR facilitates DNA end resection and HRR During HRR, the DSB is recognized by the MRN complex, which subsequently recruits BRCA1-activated CtIP to initiate short-range DNA end resection. Processed ends are further resected by EXO1 and BLM/DNA2, creating long 3′ overhang ssDNAs coated by RPA. RPA-ssDNA and CDK18 activate ATR, which in turn maintains the abundance of resection factors through E2F-mediated transcription. In the resection stage, ATR phosphorylates CtIP and EXO1. ATR-binding scaRNA2 coordinates and bridges resection factors to ATR activation. Next, RAD51 nucleoprotein filaments are assembled with the help of BRCA2-PALB2 and BRCA1. ATR is stabilized by lncRNA ANRIL and promotes HRR by phosphorylating these key HR proteins. RAD51 filaments invade the complementary DNA template to finalize faithful DSB repair. (The figure was created in BioRender).

In addition, loss of the long non-coding RNA (lncRNA) ANRIL, a novel ATR stabilizer that protects against ATR ubiquitination-mediated degradation, can lead to impaired HR.^46^ BRCA1 functions in HR are known to be bypassed during the acquisition of PARPi resistance; ATR inhibition can disrupt BRCA1-independent RAD51 loading to DSBs and overcome such resistance mechanisms.^47^ In line with these findings, Ning et al.^48^ identified ATR as a central mediator in the development of HR-related PARPi resistance in non-Myc (myelocytomatosis oncogene) glioblastoma stem-like cells (GSCs). In this setting, owing to the absence of transcriptional repression by Myc, CDK18 activates ATR via ATR-ETAA1 and ATR-TOPBP1 interactions and promotes HR and PARPi resistance. In contrast, Myc-amplified GSCs exhibit increased PARPi sensitivity. Most important, regardless of Myc status, ATR inhibition effectively sensitizes GSCs to PARPis (Figure 2).

The evidence summarized above highlights the crucial role of ATR in activating components of the HR machinery during the post-resection stage. However, recent research points to the involvement of ATR in regulating DNA end resection as well. As noted above, the abrogation of 53BP1, or the shieldin complex, can restore HR functionality. These elements, which act as negative regulators of HR, are often called end protectors in the context of DSB processing. They accumulate at break sites and bind to ssDNA to oppose end resection.^49^^,^^50^ This mechanism of PARPi resistance is characterized by the reacquisition of DNA end resection.

ATR regulates the abundance of central resection factors, including C-terminal binding protein interacting protein (CtIP), BRCA1, and BLM, through E2F-mediated transcription.^51^ ATR is also implicated in the phosphorylation of CtIP and EXO1. As such, ATR-dependent phosphorylation of CtIP is indispensable for stable CtIP chromatin binding and resection,^52^ while the phosphorylation of EXO1 by ATR restrains its activity and suppresses overresection.^53^ Recently, the novel HR promoter small Cajal body-specific RNA2 (scaRNA2) has been identified as a bridge between ATR activation and DNA end resection. scaRNA2 is characterized as the top-enriched lncRNA that binds ATR at its N- and C-terminal regions. Knockdown of scaRNA2 significantly impairs ionizing radiation (IR)-induced resection and ATR phosphorylation levels, decreasing the recruitment of MRE11, EXO1, and ATR to DSB sites. Additionally, scaRNA2 connects RPA-ssDNA to the assembly of the MRE11-Rad50-Nbs1 (MRN) complex and the activation of ATR^54^ (Figure 2). These findings underscore the central role of ATR in HR, where it either directly influences or serves as an intermediary platform governing the core processes in this repair pathway. Importantly, inhibiting ATR enhances the effectiveness of PARPis, demonstrating its potential impact of ATR beyond traditional HR pathways.

#### Coordinating cell-cycle timing

In response to DNA damage, cells engage the DDR to safeguard genomic integrity and promote survival. A central component of the DDR is the activation of cell-cycle checkpoints, which delay cell-cycle progression and allow time for DNA repair before cell progression to subsequent phases of the cell cycle. The G_1_ checkpoint blocks entry into the S phase, the intra-S phase checkpoint halts replication of damaged DNA, and the G_2_ checkpoint prevents cells with damaged DNA from entering mitosis.

The S phase checkpoint operates primarily by suppressing the initiation of new origins of DNA replication.^55^^,^^56^^,^^57^ ATR functions as an activator of the S phase checkpoint operating by phosphorylating and activating the effector kinase CHK1, which cooperates with the ataxia telangiectasia mutated (ATM)/CHK2 axis to degrade CDC25A phosphatase required for CDK2 activation, thus implementing the S phase checkpoint.^18^^,^^58^^,^^59^^,^^60^

The majority of cancers harbor a mutant p53,^61^ which results in dysfunction of the G_1_ checkpoint. Given that the S phase checkpoint is typically only a few minutes long, and thus unable to provide enough time for the completion of DSB repair,^55^^,^^56^^,^^57^ the G_2_ checkpoint gains relevance. Furthermore, DNA damage induced during DNA replication in the presence of PARPi is likely to persist in the subsequent G_2_-phase and to arrest cells at the G_2_/M border. Overall, the checkpoint activated by DSBs in this phase of the cell cycle is much longer than the S phase checkpoint and markedly facilitates DSB repair.

ATR is the principal mediator of the G_2_ checkpoint. Once activated, ATR activates CHK1, which then degrades the CDC25C phosphatase. This degradation prevents CDC25C from removing inhibitory modifications on the CDK1/cyclin B complex, thereby halting the G_2_/M transition.^18^^,^^58^^,^^59^^,^^62^^,^^63^ A novel factor nuclear factor erythroid 2-related factor 2 (NRF2) was recently discovered assisting ATR activation during this process. NRF2 harbors an ATR-activating domain similar to TOPBP1 and co-localizes with ATR at the DSB site. Indeed, depletion of NRF2 impairs phosphorylation levels of the ATR-CHK1-CDK1 axis, as well as the G_2_ checkpoint effectiveness.^64^^,^^65^

**Figure 3:**
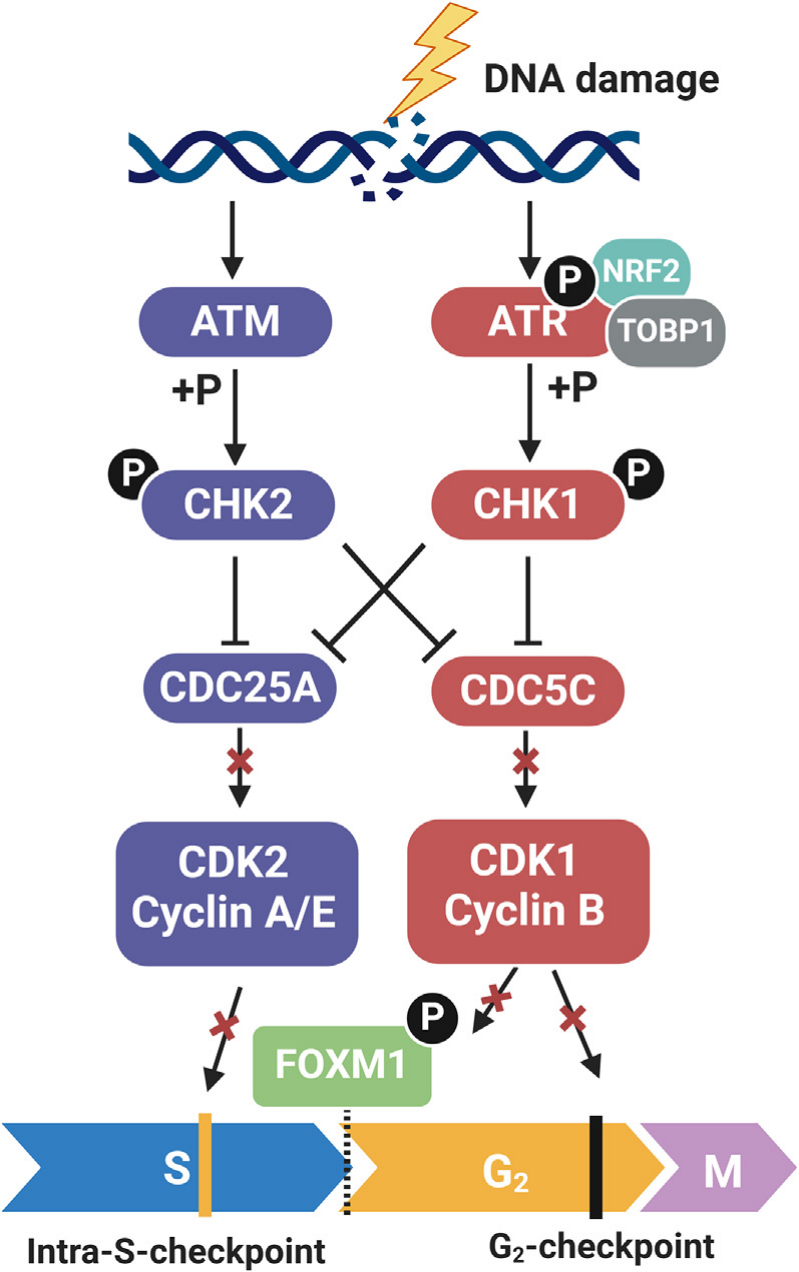
ATR regulates the intra-S and G_2_ checkpoints, as well as the S/G_2_ transition ATR acts through its downstream effector CHK1, which induces the degradation of CDC25A in S phase and of CDC25C in G_2_ phase. Both phosphatases remove inhibitory modifications from CDKs to implement intra-S and G_2_ checkpoints. ATR also blocks FOXM1 phosphorylation by CDK1 in S phase, preventing premature G_2_ entry before completed replication. (The figure was created in BioRender).

Saldivar et al.^66^ revealed an additional checkpoint enforced by ATR that regulates the S/G_2_ transition. In S phase, ATR represses the CDK1-dependent phosphorylation switch of FOXM1 (forkhead box M1), a transactivator of a mitotic transcription program, which facilitates G_2_ entry upon completion of DNA replication. ATR inhibition results in premature cyclin B accumulation in S phase and consequently early mitosis.^66^ In line with this observation, CDK1 inhibition that delays mitotic entry was found to alleviate the incidence of aberrant mitoses and genomic instability upon ATR inhibition^67^ (Figure 3).

Overall, ATR regulates cell-cycle checkpoints that respond to DNA damage generated following PARPi treatment. It coordinates DNA replication with G2/M progression, ensuring faithful genome duplication and chromosome segregation. ATR inhibition shortens the time for DSB repair, allowing damaged DNA to replicate. DSBs generated during S phase accumulate and when cells escape the G_2_ checkpoint and enter mitosis, leading to mitotic catastrophe and cell death that enhances PARPi efficacy.

The mechanisms by which cancer cells evade therapeutic interventions and develop resistance to PARPi are complex. Indeed, drug resistance is often a consequence of activation or inactivation of multiple mechanisms and pathways, rather than of a single pathway adaptation. This is the case also for resistance to PARPi. For instance, the upregulation of *ABCB1* transporter, which encodes a p-glycoprotein efflux pump, reduces the effectiveness of PARPi by increasing drug efflux.^68^^,^^69^ The HMGB3 (high-mobility group box 3), previously associated with tamoxifen resistance,^70^ was recently identified as a PARP1 interaction partner that facilitates PARP1 escape from the trapped state, thus promoting PARPi resistance.^71^ Hypoxia, a hallmark of the tumor microenvironment, can also contribute to PARPi resistance by reducing reactive oxygen species-induced DNA damage.^72^ These resistance mechanisms likely develop alongside the ATR-related mechanisms discussed above, highlighting the importance of expanding drug combinations to effectively combat this resistance.

### Preclinical and clinical studies using ATR inhibitors in conjunction with PARPi

#### OC

OC has the distinction of being the first malignancy for which PARP inhibitors were approved for HR^−^ cancers, marking a pivotal advancement in precision cancer therapy. It is noteworthy that many of the resistance mechanisms associated with PARPis were initially identified in the context of OC. Substantial advancements have been noted recently in the development of combination therapies aimed at re-sensitizing OCs to PARPi. A study by Kim and colleagues^16^ established PARPi-resistance models by continuously (∼1.5 years) treating BRCA mutant OC cell lines with olaparib. Interestingly, whole-genome sequencing data revealed that the acquired PARPi-resistant OC cell lines gained mutations in genes involved in non-homologous end joining (NHEJ), such as *PRKDC* (splice variant; encoding DNA-dependent protein kinase catalytic subunit [DNA-PKcs]) and *XRCC6* (loss of heterozygosity; encoding Ku70). This supports the notion that HR restoration may derive from re-acquisition of resection through canonical (c)-NHEJ inactivation, since NHEJ competes with HR in DSB end processing and Ku suppresses DNA end resection.

In addition, the phosphorylation of ATR and CHK1 markedly increased in PARPi-resistant OC cell lines. Based on this observation, the efficacy of the ATR inhibitor ceralasertib (AZD6738) in combination with olaparib was evaluated in several independent studies using OC cell lines. Ceralasertib was shown to strongly synergize with olaparib, enhancing OC killing by 50%–80%. This synergistic effect could be observed not only in BRCA mutant cells but also in wild-type and olaparib-resistant cells.^16^^,^^73^^,^^74^ In the latter cells, ATRi dysregulated a panel of DDR proteins (Ku70/80, MDM2, and p21), exacerbated replication stress, and overrode the cell-cycle arrest induced by olaparib, leading to chromosomal aberrations and apoptosis.^16^^,^^73^^,^^74^^,^^75^ The robustness of this combined protocol was further validated *in vivo*. In germline BRCA2 mutant PDX (patient-derived xenograft) models, the combination of olaparib and ceralasertib achieved an overall response rate (ORR) of up to 71.4%.^76^ In PARPi-resistant tumors, the combined olaparib/ceralasertib treatment resulted in near-complete tumor regression, improving median overall survival (OS) >5-fold compared to olaparib alone.^16^

The ongoing clinical trial CAPRI (this study was registered at ClinicalTrials.gov: NCT03462342), which combined ceralasertib and olaparib, provided clinical evidence for effectiveness in recurrent high-grade serous OC (HGSOC). This single-arm, phase 2 trial divided treatment cohorts based on HR status and response to platinum or PARPi. Interim outcomes of the cohort enrolling platinum-resistant/PARPi-naive patients were reported. Among the 12 patients evaluated for treatment effectiveness, none exhibited objective responses, 9 patients achieved stable disease (SD) and 3 experienced progressive disease (PD). Better response was observed in 3 patients who had BRCA1 mutations, with a higher median progression-free survival (PFS) time of 8.6 months compared to 4.2 months overall.^77^ The combined efficacy in the cohort that had acquired PARPi-resistant disease was also reported. Eligible patients had recurrent, platinum-sensitive BRCA1/2 or other HR repair (HRR) genes mutated and clinically benefited from PARPi. Of the patients enrolled in the trial, 13 were analyzable for safety and 12 for efficacy. There were 6 partial responses (PRs), yielding an ORR of 50%, and the median PFS was 7.5 months.^78^ Importantly, adverse events (AEs) possibly related to treatment were primarily of grade 1 or 2, and no patient discontinued treatment due to toxicity^77^^,^^78^ (Table 1).

**Table 1:** Summary of clinical trials on PARPi/ATRi combinations with available outcomes

Clinical trial identifier	Treatment	Trial phase	Eligible patients	Biomarkers	Clinical results	Notable AEs	References
CAPRI (NCT03462342)	olaparib + ceralasertib	2	cohort B: platinum-resistant, PARPi-naive HGSOC patients (*n* = 14); cohort C: recurrent, platinum-sensitive, HRD, and PARPi-resistant HGSOC patients (*n* = 13)	BRCA1/2	cohort B: 9 SD, 3 PD, median PFS 4.2 months; cohort C: 6 PR, 6 SD, estimated median PFS 7.43 months	cohort B: G3 nausea (14.3%), fatigue (7.1%), anorexia (7.1%), and anemia (7.1%); cohort C: G3 anemia (15%), G3 thrombocytopenia (23%), G4 neutropenia (8%)	Shah et al.^77^ and Wethington et al.^78^
OLAPCO (NCT02576444)	olaparib + ceralasertib	2	patients with cancers harboring deleterious mutations in HR genes and other DNA repair pathway genes: PARPi-resistant HGSOC (*n* = 7), PDAC (*n* = 6), CRPC (*n* = 3), ACC (*n* = 2), breast cancer (*n* = 2), sarcoma (*n* = 1), cholangiocarcinoma (*n* = 1); colorectal cancer (*n* = 1); primary peritoneal (*n* = 1); paraganglioma (*n* = 1)	BRCA1/2, PALB2, ATM, CHK2, and TCA cycle genes implicated in HR defects	HGSOC: 1 PR, 5 SD, 1 PD; PDAC: 2 SD, 3 PD; CRPC: 3 SD; breast cancer: 1 CR, 1 PD; ACC: 2 SD; colorectal cancer: 1 SD; peritoneal 1 PD; cholangiocarcinoma: 1 PD	G3 anemia (12%), G3/4 thrombocytopenia (8%), G3/4 neutropenia (12%)	Mahdi et al.^79^
SUKSES-N2 (NCT0328607)	olaparib + ceralasertib	2	relapsed, biomarker unselected SCLC patients (*n* = 21)	BRCA1/2, ATM, MRE11, RAD51, PALB2, BLM, CHK2, RAD54	PFS 2.75 months, OS 7.18 months, DCR 42.3%.	G3 thrombocytopenia (7.7%), anemia (15.4%), neutropenia (3.8%), nausea (3.8%), headache (3.8%), dyspnea (3.8%); G4 thrombocytopenia (3.8%); G5 neutropenic fever (3.8%).	Park et al.^80^
NCT02937818	olaparib + ceralasertib	2	platinum-refractory extensive-stage SCLC patients (*n* = 21)	–	PFS 2.92 months, OS 7.56 months, DCR 38.1%	G3/4 anemia (19.05%), thrombocytopenia (4.76%), asthenia (4.76%), pyrexia (4.76%), pneumonia (4.76%), urinary tract infection (4.76%)	–
ATARI (NCT04065269)	olaparib + ceralasertib	2	relapsed carcinosarcomas (*n* = 8), endometrioid carcinoma (*n* = 11), cervical cancer (*n* = 10)	ARID1A	ORR 24%, DCR 52%, PFS 23.9 weeks	39% G3^+^ toxicities	Banerjee et al.^81^
TRAP (NCT03787680)	olaparib + ceralasertib	2	CRPC patients with (cohort 1) or without DNA repair defects (cohort 2)	BRCA1/2 and ATM	cohort 1: 40% response rate; cohort 2: 14.3%	not reported	Reichert et al.^82^

ACC, adenoid cystic carcinoma; CRPC, castration-resistant prostate cancer; PDAC, pancreatic ductal adenocarcinoma; TCA, tricarboxylic acid.

The OLAPCO trial (this study was registered at ClinicalTrials.gov: NCT02576444) also investigated the combination of olaparib and ceralasertib in PARPi-resistant (acquired or *de novo*), platinum-resistant BRCA-mutated patients,^79^ applying the same dose regimen as that used in the CAPRI trial.^77^ For the HGSOC cohort, 7 patients had received 1–3 prior PARPi-based regimens and had progressed during their most recent PARPi exposure; the ORR was 14% and the SD rate was 71%, leading to a clinical benefit rate of 85.7%. Strikingly, the duration of benefit in these patients from combined olaparib/ceralasertib treatment exceeded the initial duration of response to PARPi (median 8 months vs. 4 months). In terms of AEs, 2 patients experienced grade 3/4 anemia or neutropenia and required dose reductions.^79^ These findings indicate that ceralasertib effectively restores sensitivity to olaparib in PARPi-resistant HGSOCs, with a toxicity profile that is manageable. Re-introducing PARPi therapy in patients who have developed resistance is recommended, therefore, when combined with ATR inhibition (Table 1).

Although adverse hematologic toxicities were generally reversible, the dose and duration of ceralasertib in combination with olaparib were actually compromised during these trials. To overcome this limitation, the modular, phase 1 trial D5330C00004 (this study was registered at ClinicalTrials.gov: NCT02264678) was designed and is now recruiting.^83^ The protocol replaced olaparib in this combined regimen with saruparib (AZD5305), a next-generation, highly selective inhibitor of PARP1. Of note, saruparib has shown reduced hematological toxicity when compared to olaparib in preclinical models^84^ and was well tolerated in an ongoing phase 1 study (PETRA; this study was registered at ClinicalTrials.gov: NCT04644068) of patients with HRD advanced breast, prostate, pancreatic, or OC.^85^ Therefore, combining ceralasertib with saruparib is anticipated to enhance the therapeutic index and improve the existing safety profile.

#### Lung cancer

Lung cancer stands as the predominant cancer type globally, with cancer-related deaths surpassing those of any other neoplastic disease. Advances in genome sequencing have revealed the complex genomic landscape of lung cancer, revealing numerous targetable genetic vulnerabilities. However, HRD remains infrequent in non-small cell lung cancer (NSCLC), particularly within the epidermal growth factor receptor-mutated subset (∼7%).^86^ Current data supporting the use of PARPi in NSCLC treatment are insufficient owing to the failure of many clinical trials to meet their primary endpoints.^87^ In contrast, SCLC exhibits a higher prevalence of HRD mutations (∼35%) and elevated expression of PARP1 compared to NSCLC.^88^^,^^89^ Despite hints of promising therapeutic benefits for PARPi, SCLC typically shows only a transient response to treatments, as most patients relapse with resistant disease between 3 and 6 months after the completion of initial chemotherapy.^90^ These factors pose challenges for the widespread application of PARPi, but they also open opportunities for ATRi combination in these settings.

Preclinical studies demonstrated that ATR inhibition using VE-821, ceralasertib, or M1774 was synergistic with olaparib or talazoparib in suppressing the growth of various human lung cancer cell lines and xenografts, including A549, H460, H23, H82, and H146, as indicated by a greater than or equal to log-fold decrease in the half-maximal inhibitory concentration.^91^^,^^92^^,^^93^ Chromosomal aberrations, apoptotic death, and unscheduled DNA replication events were substantially increased in cancer cells that have undergone PARPi + ATRi treatments compared to either agent alone.^91^^,^^92^^,^^93^ These combined effects were tested further in the setting of radiotherapy. Tran Chau et al.^94^ showed additive radiosensitization by combining olaparib and ceralasertib in LL2 cells and subcutaneous grafts.

To date, two phase 2 clinical trials investigated the combined effects of ceralasertib and olaparib in SCLC patients. The SUKSES study is a biomarker-driven umbrella trial that enrolled and allocated relapsed SCLC patients based on their genomic alterations. Patients with mutations in HR genes (not limited to BRCA1/2, ATM, or MRE11) were allocated to the olaparib monotherapy arm (*n* = 15, SUKSES-B; this study was registered at ClinicalTrials.gov: NCT03009682). Biomarker-unselected patients were allocated to the olaparib + ceralasertib arm (*n* = 21, SUKSES-N2; this study was registered at ClinicalTrials.gov: NCT0328607).

In the olaparib monotherapy arm, the disease control rate (DCR) was 33.3%, median PFS was 1.3 months, and median OS was 8.6 months. Of note, in the combination arm, tumor control was markedly improved (DCR 42.3%, median PFS 2.8 months), although the OS remained poor (7.2 months). Only 2 patients (7.7%) discontinued the combined treatment due to drug-related AEs.^80^ Consistent response data from the combination of olaparib and ceralasertib were obtained in another independent trial (this study was registered at ClinicalTrials.gov: NCT02937818). This trial enrolled 21 platinum-refractory extensive-stage SCLC patients, achieving a median PFS of 2.92 months, an OS of 7.56 months and a DCR of 38.1% (Table 1). Current clinical research focuses mainly on SCLC, and there is no evidence for NSCLC responses. The therapeutic index achieved so far in SCLC has not met the predefined endpoint, although disease stabilization was more evident in the combination treatment cohort. Further investigations are required, particularly when applying patient selection strategies to maximize efficacy.

#### Cancers of other organs

The combined application of PARPi and ATRi has demonstrated promising efficacy in preclinical studies of tumors across multiple organs. These include breast cancer,^93^^,^^95^^,^^96^^,^^97^^,^^98^ prostate cancer^99^ and pancreatic cancer,^100^^,^^101^ which may also be driven by BRCA1/2 mutations. Other cancers are biliary tract cancer,^102^ glioblastoma,^48^ and neuroblastoma,^103^^,^^104^ which are characterized by high replication stress. The approach even extends to non-solid tumors, such as acute myeloid leukemia.^105^

Besides HGSOC, the OLAPCO trial enrolled other genomically targeted tumor subsets involving patients with pancreatic ductal adenocarcinoma (*n* = 6), castration-resistant prostate cancer (*n* = 3), adenoid cystic carcinoma (*n* = 2), breast cancer (*n* = 2), sarcoma (*n* = 1), cholangiocarcinoma (*n* = 1), colorectal cancer (*n* = 1), primary peritoneal cancer (*n* = 1), and paraganglioma (*n* = 1). The best tumor response was observed in one case of breast cancer, achieving complete remission (CR) for up to 26 months. Also, one case of adenoid cystic carcinoma achieved 26 months of SD. Of note, both of these patients harbored ATM mutations. However, 38.1% of patients exhibited no treatment responses, experiencing continued tumor progression post-treatment. Moreover, the remaining patients achieved only 4–14 months of SD.^79^

In addition, the ATARI trial (this study was registered at ClinicalTrials.gov: NCT04065269) assessed the clinical activity of olaparib and ceralasertib in rare subtypes of relapsed gynecological cancers. In the combination cohort, which included 8 cases of carcinosarcomas, 11 cases of endometrioid carcinoma, and 10 cases of cervical cancer, an ORR of 24% (median duration of response [DOR] 41 weeks) and a DCR of 52% were observed, and the median PFS was 23.9 weeks. Notably, the efficacy demonstrated in this cohort was higher than the cohort receiving ceralasertib monotherapy.^81^

The TRAP trial (this study was registered at ClinicalTrials.gov: NCT03787680) explored the synergistic potential of olaparib and ceralasertib in castration-resistant prostate cancer. Participants were stratified into two cohorts based on the presence of DNA repair anomalies identified through next-generation sequencing. The cohort characterized by DNA repair deficiencies, specifically losses in BRCA2 and ATM, demonstrated a 40% response rate, evidenced by a confirmed decline in prostate-specific antigen levels of ≥50% in 4 of 10 patients, with 2 additional patients pending further evaluation. In contrast, the cohort without DNA repair defects, consisting of 21 participants, showed a markedly lower response rate of 14.3%, with only 3 patients exhibiting responses (Table 1). This differential outcome underscores the potential of targeted therapy based on genetic profiling in enhancing treatment efficacy.^82^

Current data in aggregate suggest that breakthrough discoveries from preclinical studies have yet to be translated effectively into clinical practice, as several trials failed to meet their designated primary endpoints. Biomarker analysis to identify genetic backgrounds beyond BRCA1/2 mutations (e.g., ATM loss), therefore, becomes essential for guiding the selection of patients who are most likely to benefit. This aspect is reviewed in the next section.

### Biomarker for combined PARPi/ATRi treatment

#### ATM

As a member of the phosphatidylinositol 3-kinase-related family of protein kinases, ATM serves as the apical kinase that orchestrates the comprehensive cellular responses triggered by DSBs.^58^^,^^106^^,^^107^ ATM is thought to play dual roles in DSB processing. It regulates c-NHEJ by facilitating the bridging of DSB ends,^108^ and also promotes HRR,^109^ primarily by regulating DNA end resection through the phosphorylation of the key resection factor CtIP.^110^ Additionally, ATM influences all three major cell-cycle checkpoints—G_1_/S, intra-S, and G_2_/M—via the phosphorylation of CHK2 and subsequent downstream signaling pathways, including those involving p53 and the CDC25A/C axis.^18^^,^^58^^,^^107^^,^^111^

ATM and ATR mediate the cell-cycle checkpoint responses to DNA damage with overlapping but non-redundant activities. Through end resection of DSBs, ATM acts as a partner to ATR in regulating cell-cycle checkpoints in a DSB load-dependent manner.^60^^,^^62^^,^^63^^,^^112^ In G_2_ cells, ATM regulates the G_2_ checkpoint epistatically with ATR under low DSB loads induced by IR.^62^ When the DSB load increases, the ATM/ATR coupling relaxes; each kinase exerts independent contributions to the G_2_ checkpoint.^62^ This crosstalk between the two kinases is also essential for efficient intra-S phase checkpoint activation.^60^ Inhibiting both ATM and ATR leads to nearly complete dysregulation of cell-cycle checkpoint control that creates vulnerabilities potentiating PARPi-based therapies.

ATM loss has been demonstrated to enhance cell sensitivity to ATRi or PARPi when used as single agents.^113^^,^^114^^,^^115^^,^^116^ A recent study reported that ATM inhibition can overcome PARPi resistance by reversing H2AX loss-driven replication fork protection in BRCA1/2-deficient cells.^117^ Such vulnerabilities are extended to settings of PARPi/ATRi combinations. For example, Neeb et al.^99^ tested rucaparib and VE-822 in prostate cancer cells and reported that ATM knockout, or ATMi-treated, cells showed hypersensitivity to combinations using both inhibitors. Gout et al.^100^ also reported strong synergy between olaparib and VE-822 in ATM-deficient and PARPi-resistant pancreatic cancer models. Such antitumor activity was further validated in PDX and organoids acquired from cancer patients with ATM loss.

Unlike ATR, which is essential in numerous organisms and whose mutations can lead to embryonic lethality,^59^ ATM is not essential for viability and is often mutated, contributing to cancer development.^107^ ATM expression, as detected by immunohistochemistry, showed a strong correlation with improved clinical outcomes in patients with muscle-invasive bladder cancer.^116^ Given its strong predictive potential, several clinical trials investigating PARPi and ATRi activities, including CAPRI, SUKSES, and TRAP, have included ATM in various DDR gene panels used for patient selection during enrollment.^78^^,^^80^^,^^82^

#### Schlafen family member 11

Schlafen family member 11 (SLFN11) is an interferon-inducible antiviral restriction factor possessing tRNA endoribonuclease and DNA binding activities.^118^ It emerges as a crucial player in preventing genomic instability by eliminating replicative damage, thus potentially serving as a tumor suppressor.^119^^,^^120^ Transcriptome analysis has revealed a robust negative correlation between SLFN11 expression and tumor sensitivity to PARPis.^121^

Resistance to talazoparib and olaparib was reported in SLFN11 knockout cell lines by Pommier’s group.^121^ This resistance stemmed from the loss of SLFN11-mediated replication reactivation block, a mechanism independent of the ATR-mediated S phase checkpoint.^119^ ATR inhibition re-sensitized SLFN11 knockout cells to talazoparib.^121^ In a subsequent study, related mechanisms were investigated further and connected to DDB1-CUL4^CDT2^ ubiquitin ligase functions.^122^ Under DNA damage stress, SLFN11 was recruited to DDB1-CUL4^CDT2^ E3 ubiquitin ligase and promoted the degradation of CDT1. Consequently, replication recovery mediated by CDT1 was irreversibly blocked, rendering cells sensitive to PARPi. Conversely, resistance was acquired when SLFN11 was inactivated. In this context, ATRi reversed cell resistance by inducing mitotic defects through CDT1 phosphorylation, which destabilizes kinetochore-microtubule attachments in SLFN11-deficient cells.^122^

More important, SLFN11 was shown to suppress the expression of ATR by cleaving type II tRNA-Leu-TAA upon DNA damage, since mRNAs encoded by the ATR gene have high TTA (Leu) codon usage.^123^ These findings support the notion that SLFN11 serves as a putative biomarker guiding the scheduling of ATRi within PARPi-based regimens.

#### Ribonuclease H2B

Ribonuclease H2B (RNASEH2B), encoded by the *RNASEH2B* gene, is one of the three subunits comprising the RNase H2 complex that removes ribonucleotides misincorporated into DNA by replicative polymerases in the ribonucleotide excision repair (RER) pathway.^124^ When RNase H2 complex is deficient, topoisomerase I outcompetes the canonical RER pathway for the processing of genome-embedded ribonucleotides, which in turn creates DNA lesions susceptible to PARP trapping.^125^ Inactivation of RNASEH2B was reported to create sensitivity of cancer cells to PARPi and ATRi.^125^^,^^126^

Zimmermann’s group^127^ introduced CRISPR-Cas9 chemogenomic screening to chart cellular factors mediating the response to a combination of RP-3500 (a novel ATRi) and niraparib. With this technology, RNASEH2B was identified together with ATM as a relevant genetic vulnerability for combined treatment protocols. This was validated *in vitro* and *in vivo* using RNASEH2B-deficient models that showed enhanced sensitivity to combinations of talazoparib/niraparib and RP-3500, albeit only when applied at sublethal doses. Mechanistically, niraparib/RP-3500 combinations generated pan-nuclear γ-H2AX and irreversible DNA replication arrest in RNASEH2B knockout cells, eventually leading to RPA exhaustion and replication catastrophe.^127^

Miao et al.^128^ expanded this research focus to concurrent genomic alterations at nearby loci on the same chromosome. Public genomic databases of pancreatic cancer revealed that *RNASEH2B* is commonly co-deleted with retinoblastoma 1 (*RB1*) and *BRCA2*, three closely located genes on chromosome 13q. Again, deletion of RNASEH2B was demonstrated to synergize with PARP inhibition in preclinical pancreatic cancer models. Interestingly, when co-deleted with RB1, RNASEH2B-driven PARPi sensitivity was significantly compromised. Notably, additional ablation of BRCA2, or the usage of ATRi that disrupts E2F1-induced BRCA2 expression, could retrieve the sensitivity of *RNASEH2B/RB1* co-deleted cells to PARP inhibition.^128^ This highlights the necessity of comprehensive genomic tests when applying RNASEH2B for predicting efficacy.

## Summary and future directions

The prevalence of PARPi resistance is a challenge in its clinical application and prompts investigations into the underpinning mechanisms as a part of efforts to overcome it. PARPis cause DNA damage that engages the ATR pathway as a rescue mechanism, generating a mechanistic rationale for combining PARPi and ATRi: (1) ATR stabilizes stalled DNA replication forks by activating fork-remodeling enzymes, providing a stable platform for removing impeding lesions; (2) ATR facilitates DNA end resection and HR to faithfully repair replication-associated DSBs; and (3) ATR activates cell-cycle checkpoints to facilitate DSB repair before mitosis. Thus, inhibition of the ATR axis disrupts several regulatory safeguards across multiple pathways that contribute to PARPi resistance.

Synergistic effects between PARPi and ATRi have been demonstrated in preclinical models across various tumors. This combination allows the administration of lower doses for each inhibitor, while still achieving improved therapeutic responses compared to either agent used alone. The recent results regarding talazoparib/olaparib and ceralasertib summarized in this review support this potential. However, available clinical trials have shown only moderate therapeutic improvements, and overlapping toxicities between inhibitors pose challenges. As only a subset of patients with specific genomic alterations shows improved responses, there is an urgent need for the identification of predictive biomarkers to underpin patient selection strategies. Currently, several biomarker genes such as ATM, SLFN11, and RNASEH2B show promising initial success. These biomarkers are crucial in regulating the cell cycle alongside ATR or in maintaining replisome stability. With the ongoing development of novel inhibitors and the expanding identification of biomarker genes for patient stratification, the combination of PARPi with ATRi is likely to gain ground as a frontline treatment for advanced cancers.

One final important consideration is that PARPi or ATRi primarily targets proliferating cells, as their antitumor mechanisms rely on replication and HR. Currently, there is no evidence supporting efficacy against quiescent stem cells. This is exemplified by the SUKSES study, where SCLC patients showed only transient responses to PARPi + ATRi therapies. While these treatments offered greater short-term disease control, they failed to improve OS, likely due to the persistence of drug-resistant quiescent cells that drive disease recurrence and progression. Therefore, employing radioactive isotopes or nanotechnology to specifically target tumor stem cells and promote cell-cycle redistribution could represent another promising direction.

## Acknowledgments

Research activities have been funded by grants from 10.13039/501100002347BMBF (02NUK037B, 02NUK043B, and 02NUK054B), the 10.13039/501100001659German Research Foundation (DFG-IL51.10, IL51.11, and GRK1739), the German Federal Ministry for Economic Affairs (BMWi-50WB1836), and Deutscher Akademischer Austauschdienst (DAAD) project no. 57515880. X.L. would like to thank the Chinese Scholarship Council for the international scholarship support. We acknowledge support by the Open Access Publication Fund of the University of Duisburg-Essen.

## Author contributions

Conceptualization, X.L., M.S., and G.I. Validation, G.I. Software, G.I. Writing – original draft, X.L. and G.I. Writing – review & editing, A.S. and G.I. Visualization, Q.Y. and G.I. Resources, M.S. and G.I. Supervision, M.S. and G.I. Project administration, M.S. and G.I. Funding acquisition, M.S. and G.I.

## Declaration of interests

Martin Stuschke: AstraZeneca (Advisory Board Function, Research and Clinical Trials), Bristol-Myers Squibb (Advisory Board Function), Sanofi-Aventis (Advisory Board Function) and Janssen-Cilag (Advisory Board Function). Other authors declare no conflict of interest.
